# 25, 50 & 75 years ago

**DOI:** 10.1111/ans.17739

**Published:** 2022-05-09

**Authors:** Julian A. Smith

**Affiliations:** ^1^ Department of Surgery Monash University Melbourne Victoria Australia

## Twenty‐five years ago

**Usatoff V, Waxman BP. A critical evaluation of free paper abstracts accepted for the 1996 RACS Annual Scientific Congress. *ANZ J. Surg*. 1997; 67: 45–6.**

Abstracts form a major part of medical information dissemination and a measure by which papers are accepted for meetings. Concerns have been raised about the quality of abstracts presented to the Annual Scientific Congress (ASC) and second, about the validity of the term ‘scientific’ to describe this meeting. A critical evaluation was made of all free paper abstracts in general surgery from the ASC 1996, using a standard assessment process. They were judged on presentation and content. A direct comparison was made to the content of abstracts from the Surgical Research Society of Australasia (SRSA) 1995 meeting. The ASC abstracts scored 87% (6.1/7.0) for presentation but with clear deficiencies. The score of 49% (7.4/15.0) for the content of the ASC abstracts was significantly less than the score of 65% (9.8/15.0) that was attained by the SRSA abstracts when assessed on content. (Wilcoxon rank sum test, *P* <0.000002). The quality of the presentation of abstracts was adequate but could clearly be improved, especially with regard to the specific instructions to authors. The ASC abstracts were significantly less scientific in content that those of the SRSA abstracts. The criteria used to select abstracts for the ASC should be reviewed and the title of the annual College meeting should be reconsidered.

**Frydman GM, Codd CA, Cavaye D, Walker PJ. The practice of carotid endarterectomy in Australasia. *ANZ J. Surg*. 1997; 67: 103–7.**

Carotid endarterectomy (CEA) is a frequently performed surgical procedure and there are variations in the preoperative, operative and postoperative management related to this operation. Questionnaires were sent to all 191 members of the Division of Vascular Surgery, Royal Australasian College of Surgeons, and the Australasian Chapter of the International Society of Cardiovascular Surgery. The questionnaire was returned by 179 surgeons (94%). One hundred and fifty‐nine were vascular surgeons, of whom 139 perform CEA. Most surgeons reported performing more CEA than 5 years previously. Surgery for asymptomatic carotid stenosis was performed by 78% of surgeons at the time of the survey. Routine carotid angiography is performed pre‐operatively for symptomatic patients by 61% of surgeons and for asymptomatic patients by 56%. Intraoperative shunting is used routinely by 37% of surgeons, selectively by 58% and never by 5%. Arteriotomy patch closure is performed routinely by 16%, usually by 30%, rarely by 52% and never by 3%. The favoured patch material is Dacron 39%, PTFE 19%, ankle long saphenous vein (LSV) 22%, thigh LSV 18% or other materials 2%. Compared with their practice 5 years previously, arterial patch closure is used more often by 42% of surgeons, the same by 51% and less by 7%. Postoperatively, patients are nursed mainly in intensive care (34%) or a high‐dependency unit (33%). The practice of CEA by Australasian vascular surgeons reflects the recent trends reported in the world literature. Most Australasian surgeons perform CEA for asymptomatic disease. Forty percent are performing CEA on the basis of duplex scanning alone. There is a trend towards increased use of patch closure. Most patients are managed in intensive care or high dependency units.

## Fifty yers ago

**Wilson WF. Shearer's knuckles. *ANZ J. Surg*. 1972; 42:192–93.**

**Fig. 1:**
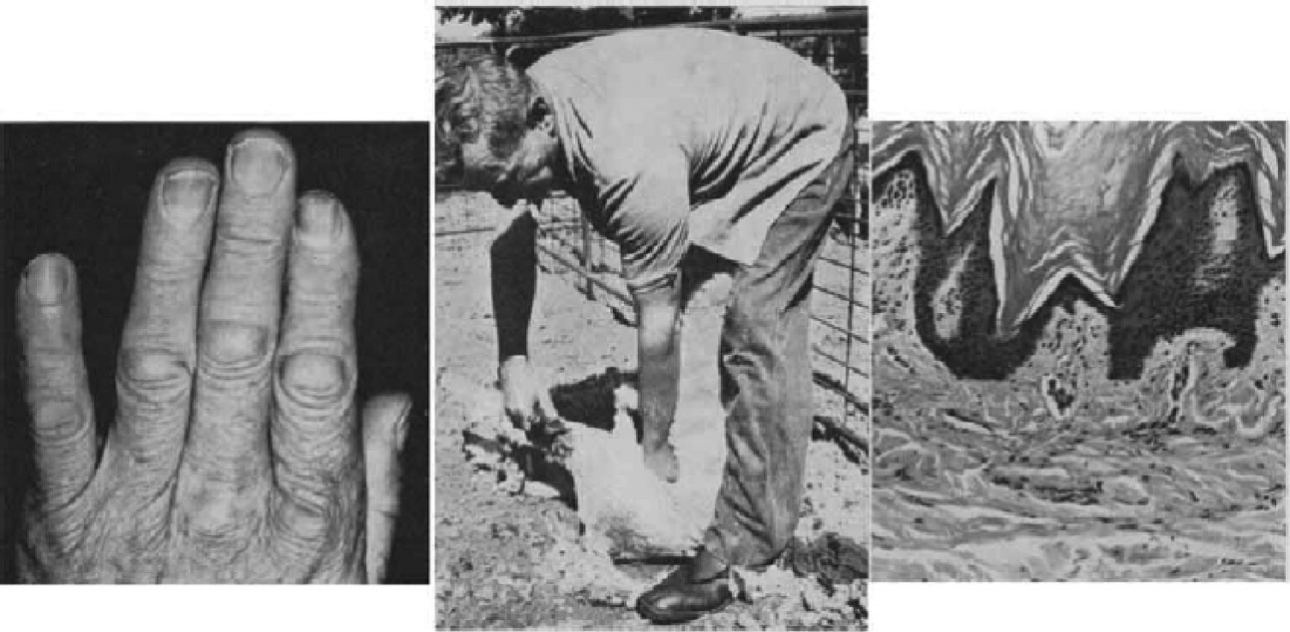
(left) Macroscopic appearance of shearer's left hand; (Centre) ‘pushing on the stifle’; (right) microscopic appearance of shearer's knuckle.

Australian shearers have always enjoyed a certain camaraderie because the distinctive appearance of their hands has provided a badge of recognition to other shearers. The commonest occupational sign Is not so much the well‐documented interdigital pilonidal sinus, but rather the appearance of dermal pads over the proximal interphalangeal knuckles on the left hand (Fig. 1, Left). Over the dorsum of the proximal interphalangeal joint on each finger of the left hand a shearer develops characteristic thickenings or knuckle pads produced by two essential positions assumed by the left hand during shearing. First, the clenched fist is used to iron out the skin and wool in front of the advancing shear, performing a similar function to the board used in advance of a hand‐knife when taking a split skin graft. Second, the clenched fist of the left hand is forced into the groin of the sheep where it is forced on to the quadriceps tendon to extend the leg for shearing, a manouvre described in the shearers' parlance as ‘pushing on the stifle’ (Fig. 1, Centre). visible change in both conditions is a loss of the transverse crease lines of the knuckles. In Dupuytren's contracture the dermis becomes stretched over the metaplastic paratendinous tissue, whereas in the shearer's knuckles the dermal hyperplasia itself causes the transverse creases to be almost erased. Thus the shearer usually shares the intemperate reputation, an Anglo‐Saxon origin and superficially similar knuckle pads with the patient with Dupuytren's contracture. The recognition of shearer's knuckles is of clinical importance because they should be distinguished from those knuckle pads which occur commonly in association with Dupuytren's contracture. The proliferative pathological process in the shearer's knuckles seems to be confined to the dermis and epidermis overlying the proximal interphalangeal joints and resembles the changes seen in callosities (Fig. 1, Right). Clinically it is interesting that the earliest shearer's knuckle pads, however, are to be regarded more as badges of honour for long service to our national industry, and this recording of their aetiology and pathology is offered to assist in the identification of a fairly typical Australian lesion.

**Windsor HM. Surgery of the mitral valve: a perspective of twenty‐five years. *ANZ J. Surg*. 1977; 42: 30–6.**

This paper considers the changing concepts in management, and the results of surgery in 1000 isolated procedures on the mitral valve, during the past 25 years. The first 251 patients were managed by closed methods with an operative mortality of 4%, but with a high reoperation rate, at least 44% ultimately having valve replacement. Open methods were first used in 1960, 17 patients having conservative valvotomy without mortality, but with indifferent results. Valve replacement began in March, 1963, and since that time 615 mitral valve replacements have been carried out, in 433 of which it was an isolated procedure. The first 290 patients had replacement with a Starr Edwards ball valve prosthesis. The actuarial curve shows a survival rate of 70% at 5 years, 61% at 8 years and probably 50% at 15 years. The remainder, except for two, had replacement with disc valves, the first 65 with Bjork Shiley, and the last 76 with Lillehei Kaster prostheses. Four patients with Bjork Shiley prostheses developed thrombosis on the prosthesis, and this was a factor influencing the change to a Lillehel Kaster prosthesis. The actuarial curve shows a survival rate of 86/% at 4 1/2 years. Since March 1963, 171 patients have had conservative procedures with preservation of the valve; in 57 it was a blind procedure and in 114 it was an open procedure, There were no early deaths in these 171 patients, but there have been four late deaths. Twenty later came to valve replacement, 12 (20%) of them being in the closed group and 8 (7%) in the open group. One hundred and twenty‐eight (74/%) are considered to be Grade I or II, most of these being in the open group.

## Seventy‐five years ago

**Wyndham N. Some aspects of the surgical pathology of the testis. *ANZ J. Surg*. 1947; 17: 47–58.**

The following is a summary of the topics presented in the article:

1. An outline of our present knowledge of testicular descent is given, showing the relationship of the gonad to the caudal end of the intermediate cell mass and its early position near the groin. No explanation of the mechanics of descent is attempted. The description is based on work a summary of which has already been published (1943).

2. Some of the salient features of cryptorchidism are outlined‐its comparative anatomy, its resultant structural and functional changes. The predisposition of such testes to malignant change is assessed. The incidence and spontaneous rectification of the abnormality lead to a consideration of the necessity of treatment.

3. Changes in hormone production consequent upon various testicular conditions are discussed. Gonadotropic hormone of the pituitary is necessary for testicular development. Abnormally high amounts of androgen are elaborated by interstitial cell tumours. Seminomata produce no hormone, but teratomata produce a luteinizing hormone. In both the last mentioned the pituitary gland produces an increased amount of follicularizing hormone. The relationship of urinary hormone assays to diagnosis and prognosis is outlined.

4. Some features of testicular tumours are mentioned‐the age periods, the ease with which such tumours are overlooked, some distinguishing characteristics of seminomata and teratomata, and the difficulties associated with adenocarcinomata.

## Acknowledgment

Open access publishing facilitated by Monash University, as part of the Wiley ‐ Monash University agreement via the Council of Australian University Librarians.

